# Adherence to cognitive and physical exercise engagement: a challenge to successful dementia risk reduction and prevention efforts

**DOI:** 10.3389/frdem.2023.1254986

**Published:** 2023-09-01

**Authors:** Therese M. O'Neil-Pirozzi, Davide B. Cappon, Alvaro Pascual-Leone

**Affiliations:** ^1^Cognitive-Community Integration Lab, Department of Communication Sciences and Disorders, Northeastern University, Boston, MA, United States; ^2^Department of Physical Medicine and Rehabilitation, Spaulding Rehabilitation Hospital, Boston, MA, United States; ^3^Hinda and Arthur Marcus Institute for Aging Research at Hebrew SeniorLife, Boston, MA, United States; ^4^Deanna and Sidney Wolk Center for Memory Health at Hebrew SeniorLife, Boston, MA, United States; ^5^Department of Neurology, Harvard Medical School, Boston, MA, United States; ^6^Institut Guttmann, Institut Universitari de Neurorehabilitació Adscrit a La UAB, Badalona, Spain

**Keywords:** cognitive exercise, physical exercise, exercise adherence, adherence assessment, mild cognitive impairment, dementia

## Abstract

With human life expectancy and proportion of older adults increasing, global use of evidence-supported preventative methods to minimize risk of brain-related disabilities such as Alzheimer's disease and other dementias—as well as interventions to slow rate of disease progression—is important. Sustained engagement in cognitive and physical exercise programs may prevent or delay dementia onset as well as maximize health and function of those with dementia. Despite awareness of the importance of cognitive and physical exercise to brain health, exercise program adherence by older adults is extremely challenging. In this Perspective article, we summarize what is known about contributors to exercise program adherence and strategies to promote it. We discuss our viewpoint on knowledge gaps regarding exercise adherence and research that needs to be conducted. We conclude by proposing a multi-dimensional exercise adherence assessment framework that includes portable neurophysiologic technologies to inform initial design and updating of individualized exercise programs that optimize sustained exercise program engagement and, ultimately, maximize brain health in older adults with and without mild cognitive impairment and dementia.

## Introduction

Human life expectancy has significantly increased in recent decades thanks to advances in public health and medicine. In developed countries, the number of older adults (>65 years) has already surpassed the number of children (<15 years), and the United Nations projects that by 2050 the proportion of elderly people will nearly double that of the young (United Nations, 2019). However, this extension of human lifespan has not translated into a parallel extension of a *healthy* lifespan (Passarino et al., 2016). Advancing age is the major risk factor for development of brain-related disability due to neurological and psychiatric diseases (Barnett et al., 2012). At present, for each added year of lifespan, 9 of 12 months are months of disability, with increasing health care expenditures exacerbating challenges associated with this trend.

Disability-adjusted life years illustrate the striking impact of brain-related disabilities (e.g., Alzheimer's disease and other dementias), accounting for 13% of the global burden of diseases (Collins et al., 2011). At present, one in four people in the world suffers brain-related disability, and according to the World Health Organization (WHO), by 2030, half of the world-wide economic impact of disability will be due to brain-related disability (Mathers and Loncar, 2006). This is a staggering challenge, and its magnitude continues to grow, affecting not only older adults with brain-related disability, but also their families, friends, and society at large. Unless we develop interventions that minimize the impact of brain-related disabilities, society will face an unsurmountable crisis.

Despite enormous efforts and substantial investments from public and private sectors, progress addressing the challenge of neurological and psychiatric disorders in older adults has been small. By the time patients become symptomatic and a diagnosis is made, impact of brain diseases is already significant and complex. Therapeutic interventions rarely achieve a cure and have limited impact on disability. This is partly because most major brain disorders start many years before symptoms manifest, and, by time of clinical presentation, intricate processes of adaptation—both of brain structure and function and of individual and social behavior—have become established. Perhaps, a primary focus should be on preventative approaches to minimize risk of development of these diseases. And for those who already have a brain disease, a major goal should be on interventions that might prevent disease progression and minimize disability. If such efforts were successful, the impact would be substantial. For example, in the case of dementia alone, delaying onset of symptoms for just 1 year could prevent disability in over 11.8 million cases in the next 30 years, representing a cost savings of $219 billion (Zissimopoulos et al., 2014).

Alzheimer's disease (AD), the most prevalent form of dementia, affects roughly 50 million people globally and is a major cause of disability (Alzheimer's Association, 2022). AD and other dementias impact patients, families, other carers, and society at large, with annual societal expenditures of $605 billion worldwide (World Health Organization, 2017). Mild Cognitive Impairment (MCI), characterized by an observable decline in cognitive abilities, is a risk factor for development of dementias. Prevalence estimates of MCI range between 16 and 20%, and incidence rates (1,000 person years) vary between 5.1 and 168, with reported progression from MCI to dementia from 20 to 40% per year (Roberts and Knopman, 2013).

The Lancet Commission on dementia reported that 12 modifiable risk factors account for 40% of worldwide dementias which could be prevented or delayed, with two of these being physical inactivity and lack of cognitive engagement (Livingston et al., 2020). Evidence supports that cognitive and physical exercise can reduce disability and, in some cases, may delay progression of dementia (Global Council on Brain Health, 2017; De la Rosa et al., 2020; Su et al., 2022; Zhu et al., 2022). Programs focused on addressing these modifiable lifestyle factors are critically needed to prevent or delay onset of dementia and, for those who already have dementia, to reduce disease-related disabilities.

Mounting evidence supports that regular engagement in challenging and purposeful cognitive exercise and physical exercise induces multiple benefits on cognitive functioning, mobility, independence, and overall quality of life (Tesky et al., 2011; Vreugdenhil et al., 2012; Global Council on Brain Health, 2017; O'Neil-Pirozzi et al., 2019). In a 2019 systematic review of computer-based cognitive intervention randomized control trials for older adults with and without MCI, it was concluded that computerized cognitive training improved cognitive functions (i.e., attention, episodic memory, processing speed, executive function, visuo-spatial functions, and working memory) in both cohorts and that timely training may delay onset of dementia and AD (Alnajjar et al., 2019). In a 2021 review of systematic reviews of physical activity intervention randomized control trials for older adults with older adults with MCI or dementia, it was concluded that physical activity interventions (e.g., aerobic exercise programs) improved overall cognition, as well as executive function and delayed recall in both cohorts (Di Lorito et al., 2020).

World-Wide FINGERS (WW-FINGERS) is an inter-professional global network committed to preventing and reducing the risk of dementia (Kivipelto et al., 2020). Their research examines efficacy of interventions (e.g., cognitive training and physical exercise) targeting *multiple* modifiable lifestyle factors. In one study, older adults at increased risk of dementia were randomized to a 2-year multi-domain intervention group consisting of cognitive training, physical exercise, nutritional guidance, social activities, and management of vascular and metabolic risk factors. Results demonstrated improved cognitive abilities, decreased risk of developing new chronic diseases and of functional decline, and increased health-related quality of life in the experimental group compared to a control group (Ngandu et al., 2015).

Thus, it is essential that older adults engage in ongoing cognitive and physical exercise to maximize brain health and function. However, adherence, defined as maintaining a regular exercise schedule after the initial adoption phase, is key to obtaining and maintaining health benefits associated with engagement in any kind of exercise program. Without sustained adherence to exercise, brain health benefits are not achievable. Despite awareness of the importance of engagement in ongoing cognitive and physical exercise to brain health and to dementia risk reduction and prevention, sustained exercise engagement is extremely challenging for older adults with and without MCI or dementia, with <50% adhering to current exercise recommendations (Tesky et al., 2011; Roberts and Knopman, 2013; Global Council on Brain Health, 2017; World Health Organization, 2017; De la Rosa et al., 2020; Livingston et al., 2020).

In this paper, we summarize current understanding of contributors to cognitive and physical exercise program adherence; discuss evidence-supported approaches to promote exercise program adherence that may be used by brain health coaches, dementia care specialists, and others; and identify knowledge gaps to encourage needed exercise adherence research. Sustained engagement by older adults in lifestyle modification approaches in general—and regarding cognitive and physical exercise in particular—may minimize disability, prevent disease progression, and positively impact families, friends, and society at large.

## Contributors to exercise adherence

Multiple factors impact cognitive and physical exercise adherence success in older adults. These factors may be grouped into five domains: (1) *personal*, (2) *behavioral/psychological*, (3) *environmental/social*, (4) *neurobiological*, and (5) *exercise program* domains. To successfully create an individualized exercise program that an older adult will adhere to—and benefit from—factors in each of these domains, some modifiable and some not, should be considered at time of initial assessment and program development and reviewed regularly over time. Ongoing awareness of non-modifiable factors and opportunity to optimize modifiable factors would enhance an older adult's sustained adherence to a beneficial exercise program and maximize their health and function.

*Personal* domain factors include age; educational level; knowledge, experience, perceptions, and beliefs regarding exercise; lifestyle preferences (active/sedentary); and awareness of exercise-related resources (e.g., educational, financial, transportation) (Room et al., 2017; Rivera-Torres et al., 2019; Saghayi et al., 2020; Cabral et al., 2022). For example, based on their 2020 systematic review and meta-analysis of exercise adherence by older adults with MCI and dementia, Di Lorito et al. (2020) speculated that adults with MCI and dementia who are over 80 years of age may benefit from more coaching to adhere to exercise programs than those 80 years of age and younger. Most, if not all, exercise questionnaires include some demographic queries. Examples include the Anamnesis and Social Determinants of Health Form (Cattaneo et al., 2018) and the Older People's Health Survey Questionnaire (New South Wales Public Health Division, 2000).

*Behavioral/psychological* domain factors include mood/mood disorders; motivation; self-efficacy; resilience; and self-regulation (Room et al., 2017; Rivera-Torres et al., 2019; O'Neil-Pirozzi, 2021; Cabral et al., 2022; O'Neil-Pirozzi et al., 2022). For example, in a systematic review of factors contributing to home-based exercise program adherence by adults 18–82 years of age, higher levels of motivation and self-efficacy, along with absence of depression and anxiety were associated with greater adherence (Bachmann et al., 2018). Many tools exist that assess behavioral/psychological factors. Examples of mood/mood disorder measures include the PHQ-9 (Kroenke and Spitzer, 2002) and GAD-7 (Spitzer et al., 2006). The BREQ-3 Physical (Markland and Tobin, 2004) and BREQ Cognitive (O'Neil-Pirozzi et al., 2022) assess motivation. Other tools appropriate for use with older adults include the Physical Exercise Self-Efficacy Scale (Neupert et al., 2009), the Cognitive Exercise Self-Efficacy Scale (O'Neil-Pirozzi, 2021), the Resilience Scale for Older Adults (Wilson et al., 2022) and the PASR-12 (Umstattd et al., 2009) for physical activity/exercise self-regulation. To date, no parallel, published, cognitive activity/exercise regulation tool exists.

*Environmental/social* domain factors include access to exercise-related resources (e.g., educational, coaching/mentoring, financial, transportation); living arrangements (alone/with others); socio-economic status; and community integration/support (Room et al., 2017; Farras-Permanyer et al., 2019; Rivera-Torres et al., 2019; Cabral et al., 2022). For example, though not statistically significant, Di Lorito et al. (2020) found that exercise adherence was higher across reviewed studies when the exercise program was in a group format and in the community. Most, if not all, exercise questionnaires include some environmental/social queries (e.g., exercise education; transportation; living arrangements; socio-economic status; community integration/support). Examples include the Home and Community Environment (HACE) Instrument (Keysor et al., 2005) and the British Geriatric Society (2019) Environmental and Social questionnaires (2019).

*Neurobiological* domain factors found to impact exercise adherence in older adults include cognitive and physical abilities, fatigue, pain, and individual differences in brain structure and function (Room et al., 2017; Tompson et al., 2018; Saghayi et al., 2020; Morris et al., 2022). For example, in an exercise adherence study of older adults with MCI by Tak et al. (2012) that was cited in the review by Di Lorito et al. (2020) referenced above, the fewer the health complaints (i.e., injuries and limitations), the greater the exercise adherence. In an umbrella review of exercise intervention adherence by individuals with chronic diseases and older adults, including those with MCI and dementia, physical and mental health status (e.g., pain and fatigue) negatively impacted exercise program adherence (Collado-Mateo et al., 2021). Many tools exist that may be used to assess cognitive and physical abilities, fatigue, and pain, all neurobiological factors that are known to influence exercise adherence by older adults. Examples include the Montreal Cognitive Assessment (Nasreddine et al., 2005), the Fullerton Functional Test (Jones and Rikli, 2002) of physical abilities, the Visual Analog Scale for Fatigue (VAS-F) (Lee et al., 1991), and the Numeric Rating Scale (Haefeli and Elfering, 2006) for pain. Physiological wearable devices (e.g., accelerometers and heart rate monitors) may be used to quantify habitual values, variability, and function over a designated period of time to inform determination of exercise dosage that will be adhered to Teixeira et al. (2021), Ferguson et al. (2022).

Although neuroimaging research regarding exercise adherence is in its infancy, age-related brain structure and function changes have been implicated in physical exercise adherence outcomes in older adults (Farras-Permanyer et al., 2019; Morris et al., 2022). For example, using structural and functional MRI, Morris et al. (2022) found that higher cortical thickness in somatosensory and inferior frontal regions; less surface area in primary visual and inferior frontal regions; higher nodal functional connectivity in default, frontoparietal, and attentional networks; and less nodal strength in primary visual and temporoparietal networks predicted older adults' physical exercise program adherence. Given their portability and cost-effectiveness, electroencephalography (EEG) and functional near-infrared spectroscopy (fNIRS) are two other neuroimaging technologies that have been used in exercise intervention outcome studies and show great potential to inform design, adherence, and success of individualized cognitive and physical exercise programs for older adults with and without MCI or dementia (Herold et al., 2018; Ji et al., 2019; Gramkow et al., 2020).

*Exercise program* domain factors also influence individuals' adherence. These include exercise challenge (degree of), enjoyment (amount of), understanding of specific exercise rationales, relation to goals and expected benefits, past exercise experience (success/failure), and ongoing program review/updating (O'Neil-Pirozzi and Hsu, 2016; Hobson et al., 2019; O'Neil-Pirozzi et al., 2019; O'Neil-Pirozzi, 2021). For example, in the review by Collado-Mateo et al. (2021), factors key to exercise adherence included exercise frequency, duration, and intensity; exercise enjoyment; and ongoing exercise program education and goal setting. One published tool that assesses these factors is the Preference for and Tolerance of the Intensity of Exercise Questionnaire (Ekkekakis et al., 2005). Informal interviews, questionnaires, and surveys may also be used to: (a) prospectively explore the potential impact of these factors when developing exercise programs and (b) continuously explore facilitators and barriers to individuals ongoing exercise program adherence.

## Strategies to promote exercise program adherence

Many evidence-supported approaches to promote exercise program adherence exist that may be used by brain health coaches, dementia care specialists, and others to promote brain health and function in older adults with and without MCI and dementia. As an extension of contributors to exercise adherence summarized in the previous section, the review by Collado-Mateo et al. (2021) identified 14 factors most frequently suggested across 55 articles to increase physical exercise adherence by individuals with chronic diseases and older adults. And, the review by Di Lorito et al. (2020) identified several evidence-supported strategies across 41 articles to increase exercise adherence by older adults with MCI and dementia. Summarized by domain, these include: (1) *personal*, (2) *behavioral/psychological*, (3) *environmental/social*, (4) *neurobiological*, and (5) *exercise program* strategies. The Table 1 provides sample evidence-supported strategies per exercise adherence domain.

**Table 1:** Evidence-supported strategies per exercise adherence domain.

**Exercise adherence domain**	**Some evidence-supported exercise adherence strategies (Di Lorito et al., 2020; Collado-Mateo et al., 2021)**
Personal	1) Education regarding exercise benefit 2) Education regarding exercise “how-to” 3) Exercise preference identification
Behavioral/psychological	1) Confidence building regarding achieving exercise success 2) Individualized, collaborative goal setting 3) Motivational interviewing
Environmental/social	1) Coaching 2) Exercise reminders (e.g., via phone calls, wearable tracking devices) 3) Positive reinforcement for effort and success
Neurobiological	1) Consideration of current cognitive abilities 2) Consideration of current physical abilities 3) Impact of exercise engagement on fatigue and pain
Exercise program	1) Appropriately challenging and enjoyable exercise activities 2) Ongoing program monitoring/adjustments (e.g., via participant feedback and smart tracking technologies) 3) Regularly scheduled exercise routine

## Knowledge gaps in exercise adherence research

Further research is needed to: (1) better understand determinants of exercise adherence and (2) identify individualized methodologies to optimally promote exercise adherence in older adults with and without MCI and dementia.

Characteristics in personal, behavioral/psychological, environmental/social, neurobiological, and exercise program domains have all been reported to influence exercise adherence in older adults. What is not yet well-understood is the relative impact of each characteristic in each of these five domains across older adults with and without MCI and dementia. We posit that multiple exercise adherence profiles exist that vary in relative weighting of contributors within and across domains to exercise adherence across older adults. For example, in pilot exercise adherence data collected by the current authors, one person with MCI was aware of their need for exercise reminders but did not believe that they would use them successfully (+executive function/–self-efficacy), while another wrongly believed that they would remember to exercise without reminders (–self-efficacy/–executive function). Based on these differing profiles, strategies to empower each to adhere to an exercise program would vary.

**Figure 1:**
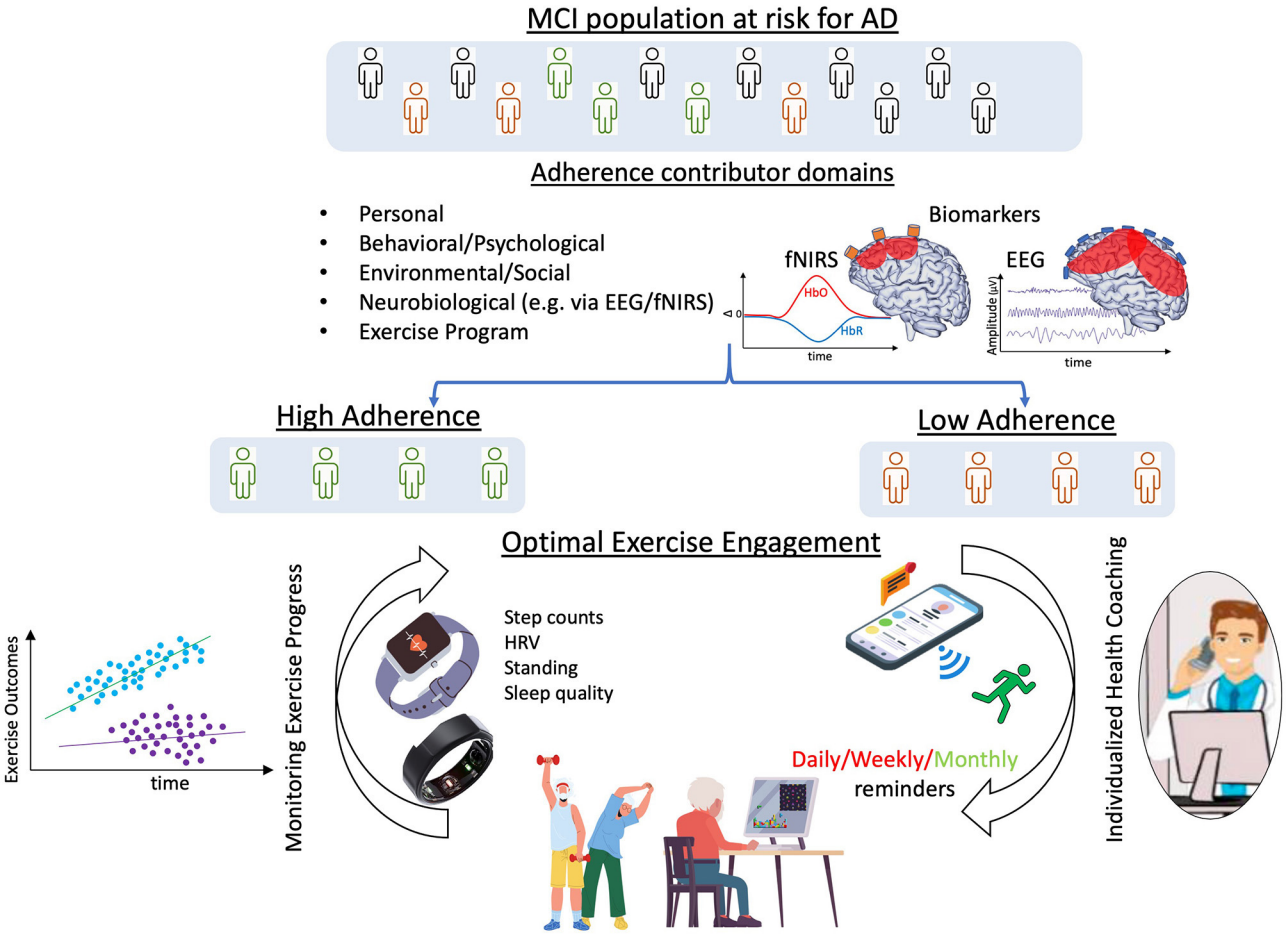
Framework for optimization of adherence to cognitive and physical exercise. By considering all factors influencing adherence and using neurophysiological biomarkers to distinguish between individuals with low and high levels of adherence, exercise programs, coaching styles, and frequency of health coaching interactions could be customized. Additionally, use of smart devices, like watches and rings, would enable data gathering and adherence monitoring, eventually further optimizing exercise programs to enhance adherence. *Made with Canva*^®^ *Version 2.0, 2021* ©.

Specifically regarding the *neurobiological* domain, we believe that research into use of EEG and fNIRS technologies with older adults to assess presence/absence of cortical markers known to correlate with or predict exercise adherence would inform the design of beneficial, individualized, cognitive and physical exercise program prescriptions (e.g., health coach, messaging). Measuring neurophysiologic adherence biomarkers as part of a baseline assessment protocol across domains, we may be able to distinguish between people with low and high levels of adherence, allowing us to customize exercise programs, health coaching methods, and frequency of exercise coaching interactions (Figure 1). Additionally, use of smart devices like watches and rings would enable ongoing data gathering and adherence monitoring, further optimizing individuals cognitive and physical exercise programs. Moreover, repeated EEG and/or fNIRS measures would inform timely exercise program adjustments that would lead to sustained adherence and continued brain health benefits.

There are many unanswered questions about *Exercise program* domain factors and their influence on exercise adherence by older adults with and without MCI and dementia. For example, how much adherence to an exercise program is needed to obtain and/or maintain benefit from it? Is 3 days' completion of a recommended 5-day/week exercise program as beneficial as 4 days' completion? Is 45 min daily completion of a recommended 60-min daily exercise program as beneficial as 60 min? With some research suggesting that reduced levels of exercise program adherence may still be beneficial, continued research regarding dosage decisions is needed.

While many current dementia prevention interventions are multi-modal in nature [e.g., WW-FINGERS (Ngandu et al., 2015; Kivipelto et al., 2020)], examination of the individual impact of each intervention component on brain health is challenging. Furthermore, it is not known if a person's adherence—and/or strategies to promote adherence—across multiple interventions is constant or varies. For example, factors that influence adherence to cognitive exercise may be different from factors that influence adherence to physical exercise. Controlled studies of uni-modal intervention adherence compared with multi-modal intervention adherence would inform individualized intervention design and thereby maximize brain health outcomes within and across older adults with and without MCI and dementia.

## Conclusion

Given the growing numbers of persons with MCI and dementia globally and the evidence that cognitive engagement and physical activity can prevent/delay the risk of dementia, older adults' engagement in individualized cognitive and physical exercise programs is important. Well-documented adherence challenges to continued exercise engagement need to be more deliberately addressed than they have been to date. We endorse the value of utilizing an individualized, multi-dimensional assessment framework of adherence consisting of personal, behavioral/psychological, environmental/social, neurobiological, and exercise program domains that fosters common practices of cognitive and physical exercise prescriptions and is followed by ongoing assessment of older adults' program adherence and brain health benefits in addition to warranted exercise prescription adjustments. More research is needed that focuses on improving exercise program adherence and ensuring that adherence to cognitive and physical exercise by older adults with and without MCI and dementia also benefits their brain health.

## Data availability statement

The original contributions presented in the study are included in the article/supplementary material, further inquiries can be directed to the corresponding author.

## Author contributions

TO'N-P: Conceptualization, Investigation, Methodology, Project administration, Resources, Visualization, Writing—original draft, Writing—review and editing. DC: Conceptualization, Resources, Visualization, Writing—original draft, Writing—review and editing. AP-L: Conceptualization, Writing—original draft, Writing—review and editing.
